# Relationship Between Implant Connection and Implant Fracture: Systematic Review

**DOI:** 10.3390/bioengineering12040333

**Published:** 2025-03-23

**Authors:** Ignacio Fernández-Asián, Daniel Torres-Lagares, María-Ángeles Serrera-Figallo, José-Luis Gutiérrez-Pérez

**Affiliations:** 1 Department of Anatomy, Faculty of Medicine, University of Seville, 41009 Seville, Spain; 2 Department of Stomatology, Faculty of Dentistry, University of Seville, 41009 Seville, Spain; danieltl@us.es (D.T.-L.); jlgp@us.es (J.-L.G.-P.)

**Keywords:** biomechanical analysis, dental implant, implant fracture, prosthetic connection, finite element analysis

## Abstract

The design of dental implants has undergone minor modifications over the years to reduce possible future complications that may arise from implant rehabilitation. One critical element from a clinical point of view is the implant connection. Given this, the team of authors of the present study decided to biomechanically analyze the effect of implant connection obtained in a possible implant fracture through a systematic review of the published literature. To this end, a search was conducted in the PubMed and Scopus databases. Only finite element studies carried out in vitro and simulation studies were included, discarding clinical studies and related reviews. A total of 19 studies were obtained for analysis and complete study. The conical type is the connection design that demonstrates the best biomechanical behavior. However, there was also significant heterogeneity in the design of the studies, which translates into a substantial source of bias, as well as different types of specific designs within the conical connection. In conclusion, it was established that the design of the connection seems to play a fundamental role in the fatigue resistance of the implant when subjected to load. However, more studies are needed to determine the most optimal specific design.

## 1. Introduction

Implantology has undergone a series of modifications over the years, both from a surgical and prosthetic point of view. These modifications, along with research accompanying this branch of dentistry in recent decades, have contributed to dental implants becoming a common therapeutic resource in clinical practice, fundamentally due to their high success rate, between 90–95% after 10 years of rehabilitation treatment [1,2].

On the other hand, despite its high success rate, implantology is not without complications. These complications can affect implant rehabilitation either from a biological perspective (6–43%) (peri-implantitis, non-osseointegration, tissue alterations, etc.) or from a mechanical perspective (9.5–49%) (damage to some components of the rehabilitation) [3,4,5].

Among biomechanical complications, one of the most important is the fracture of the implant itself. This event irreversibly compromises the rehabilitation, as treating this complication requires the implant to be surgically removed or omitted as a rehabilitation pillar [5,6].

For this reason, the design of implants (surface, connection, thread design, etc.) has been altered with slight modifications over the years to reduce possible complications that may occur in implantological rehabilitation. One of the most clinically significant elements is the implant connection. Originally, endosseous implants were made with an external connection. However, due to a series of biomechanical and biological complications associated with these rehabilitations, the implant connection type was modified and an internal connection was designed [7,8]. Despite this, several authors advocate that each type of connection has a series of strengths and weaknesses compared to the other [9,10].

In addition, within the internal connection, different geometric figures can be found in the design of endosseous implants (hexagonal, tetralobed, conical, etc.). Because of this, clinicians may struggle to recognize which type of connection may present better characteristics from a biomechanical point of view in the face of the burden of rehabilitating a patient. It is true that, when searching for published studies, they may find a very heterogeneous design and comparative analyses between different designs of different connections.

Due to all this, the team of authors of the present study decided to biomechanically analyze the effect obtained by implant connection on a possible implant fracture through a systematic review of the published literature. The hypothesis was that the internal cone-type connection would be the one that would present the best behavior. However, due to the great diversity of designs available on the market, the objective was to make an analysis of all the finite element studies published to date of different connections to establish which design best reacts to the load and if it plays a fundamental role in implant fatigue resistance.

## 2. Materials and Methods

A systematic review was performed following the indications of the Preferred Reporting Items for Systematic Reviews and Meta-Analyses (PRISMA) statement [11]. This review aimed to answer the PICO (Patient/Population, Intervention, Comparison, and Outcome) question: Does the implant connection design influence the possible fracture of the implant when it is subjected to loading?

The analyzed population included different designs of dental implants. In addition, the intervention was determined by the various designs of the implantological connection. The related comparison is the type of load suffered by the rehabilitation, and the result was a fracture of the dental implant.

The PubMed and Scopus databases were used for the search (grey literature was not included in this study), and the following algorithm was used: “Dental Implant AND (Connection OR Prosthetic Connection) AND (Finite Elements OR FEM) AND (Implant Fracture OR Implant Failure).”

This systematic review was prepared using resources provided by the University of Seville (Spain). Two researchers collected information on the study design, the type of implant connection analyzed, and the resulting failure rate for each connection.

This was intended to cover everything published to date on finite element studies analyzing implant fractures and their prosthetic connection.

The inclusion criteria established were as follows:

- Articles published to date (01-11-25).
- Articles published in English.
- Studies based on finite elements.
- Articles relating to dental implant fractures and their connection characteristics.
- Only simulations or in vitro studies were included.

On the other hand, there were reasons for exclusion from the present review:

- Articles published in non-English languages.
- Studies unrelated to finite element tests.
- Studies that did not relate implant fracture to prosthetic connection.
- Reviews were discarded.

## 3. Results

**Figure 1:**
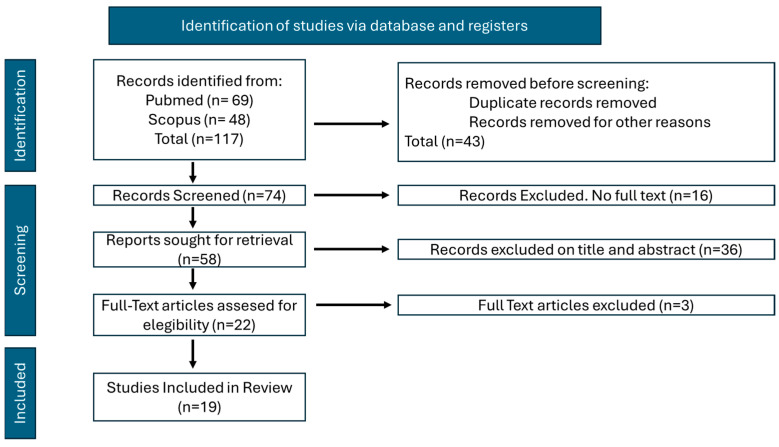
Review flowchart.

The search in both databases (PubMed and Scopus) obtained 117 studies, which were ultimately reduced to 19 studies once the aforementioned inclusion and exclusion criteria were applied (Figure 1). For the articles obtained and analyzed in full text, some of the corresponding authors were contacted via email to expand on some information for the review. Although the response from the authors obtained a high rate of return, no additional information could be added.

From the full-text articles analyzed, the type of study design, the sample analyzed, and the key factors related to the implant connection that compromised rehabilitation and their influence on implant fracture were observed (Table 1). The two researchers who carried out the search and data filtering first discarded duplicate articles from both databases. Secondly, they excluded studies in which the role of the connection as a differentiating factor in the resistance of the implant to the load was not analyzed.

**Figure 2:**
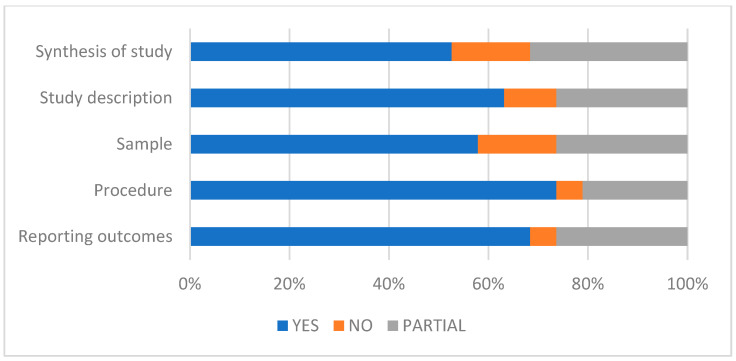
QA results of the included studies using the DARE assessment criteria.

On the other hand, an analysis of the studies included in the review was also conducted to assess their quality and the possibility of bias using the DARE evaluation criteria (Figure 2) [31].

## 4. Discussion

First, after collecting and evaluating the data obtained in the studies analyzed in this review, the researchers of the present study decided not to perform a meta-analysis due to the heterogeneity in the design of the studies.

The trial’s main objective was to analyze the influence of implant connection design on the implant when subjected to load through a systematic literature review. Currently, dental implants for rehabilitating missing teeth are used as a therapeutic resource of first choice. However, this therapeutic solution is not exempt from complications because, when placed in an environment such as the oral cavity, they must withstand a series of biological and biomechanical adversities [32,33,34].

Within these possible complications, we could consider biomechanical complications, which are fundamentally caused by the load received during implant rehabilitation. To avoid or minimize this, several modifications have been made in the design of implants over the years, along with a thorough study of the different prosthetic components from both microscopic and macroscopic perspectives [35,36,37].

The implant connection is one of these macroscopic factors modified over the years. Although different types of connections can be found on the market (external hexagonal connection, internal hexagonal, tube-in-tube, Morse taper, etc.), and all have a wide range of characteristics and are feasible for use, it seems to be established that the connection that best distributes and offers better resistance to load is the conical type [12,17,20]. Giner et al., in their study, analyzed the behavior of the conical connection compared to the tube-in-tube connection when subjected to load, obtaining statistically significant (*p* < 0.05) better results for the conical connection [16]. Similar results were presented by other authors, such as Balik et al. and Mitra et al., who conducted studies comparing the conical connection with different types of designs (hexagonal internal, hexagonal external, etc.) and obtained better results for the conical connection compared to the others [25,30].

This may be mainly because, according to the studies analyzed, the conical connection seems to provide the highest stability. This factor may determine its homogeneous transmission of forces, reducing stress concentration at specific points [12,15,16]. Lemos CAA et al. designed a study in which they analyzed the microstrain values produced after the application of mechanical load in conical connection implants (range: 1295–1432 με) versus external hexagonal connection implants (range: 1832–2715 με), obtaining lower results for the conical connection [17]. De Paula GA et al., in a simulation study, analyzed these two types of connections and the stress concentration generated by the load, obtaining a lower concentration in a given area and, therefore, a better dissipation for the conical connection [29].

On the other hand, the type of conical connection is also an essential factor to take into account. This is because, within this type of design, different cone models can be found (with greater or lesser depths of the cone, as well as various degrees of conicity). Balik A. et al., in their published study analyzing five different connection types, concluded that the connection that performed best under load was the tapered connection associated with an internal hexagon [25].

Additionally, the degree of taper seems to be a determining factor in the resistance of the implant to loading. Wang K et al., in their study, reported that a greater or lesser degree of taper may affect the thickness of the implant walls in the connection area, thereby influencing fracture resistance at this level [23]. On the other hand, Yao KT et al. argued in their study that the optimal degree of taper should be established for each type of conical connection since not all share a similar macroscopic design [21]. In their study, Yao KT et al. argue that the optimal degree of taper should be established for each type of conical connection since not all have a similar macroscopic design [21]. In their study, Yao KT et al. argue that the optimal degree of taper should be established for each type of conical connection.

The latter can represent an important source of bias. Although the inner cone appears to be the geometric figure that performs best under load, other factors, such as its depth and the degree of taper, can play a fundamental role in biomechanical behavior.

Another aspect to consider in the biomechanical resistance of the implant is its width. According to the studies published in the literature, the implant diameter seems to directly influence the load resistance of the implant [38,39]. However, some of the studies analyzed in this systematic review emphasize that the type of connection may influence this parameter to some extent. Alberti A. et al. biomechanically compared two types of implants, thin (3.3 mm) and extra-thin (2.9 mm), and obtained similar results, with no statistically significant differences (obtaining fatigue limit results at 220 and 240 N, respectively). They attributed the results to the type of connection as the main factor in the biomechanical resistance of the implant [14]. Bordin D et al., in a similar study, observed that biomechanical complications were reduced to the fracture of the prosthetic abutment, finding similar results in the resistance of the implant to fracture [22]. In a similar study, Bordin D et al. observed how biomechanical complications were reduced to the fracture of the prosthetic abutment, finding similar results in the resistance of the implant to fracture.

Another parameter that seems to play a crucial role is the platform change. This appears to dissipate the load along the implant walls more homogeneously than the regular platform, which may present a higher concentration of stresses at specific points. Liu S et al. conducted a study comparing connection implants with platform change versus regular platforms, obtaining better results for the connection presented by platform change. However, it should be noted that the implants analyzed in this study changed this parameter and other geometric and structural aspects of the implant, making it a potential source of bias, as seen in different studies [26].

Freitas-Júnior AC et al. analyzed the external versus internal hexagonal connection with and without platform change using implants from the same manufacturer and with similar macroscopic designs. Although favorable results were obtained for the internal hexagonal connection when it had a change of platform, the same was not observed for the external one. This suggests that an isolated geometric element does not have to benefit from the biomechanical point of view, but rather the design as a whole [27].

In addition to the width and geometric design of the implant, another parameter that should be considered is the length of the implant. When force is exerted on implant rehabilitation, it is distributed across all components (implant and prosthetic attachments). A larger implant size will help to dissipate the stress better. One way to increase the implant area is to increase its length. Therefore, it is logical that the load may compromise the implants with a reduced length compared to those of standard lengths. However, published studies refer to the length as a secondary element and the resulting implant width as a more critical factor than the latter [40,41]. In addition to geometric design, using surfaces and materials with better biomechanical behavior is another way to obtain a lower complication rate under loading [42,43].

For these reasons, Araki H et al., in their study, compared different implant connections lengths when subjected to a given load. They concluded that in more biomechanically compromised situations, such as in the case of shorter implant length, a Tissue Level (TL) connection type should be considered, as it appears to concentrate less stress at the bone level than the Bone Level (BL) implant [18]. Similarly, Lee H et al. conducted a study comparing different implant connections in implants of reduced length when subjected to loading. They obtained results similar to the previous study, concluding that the tissue level connection seemed to behave better biomechanically than the bone level connection, making it a viable option in compromised clinical situations, such as the use of short implants [19]. The results were similar to the previous study, in which they concluded that the Tissue Level connection seemed to behave better biomechanically than the bone level connection, so it should be an option to be taken into account in compromised clinical situations such as using short implants [19]. The results of this study were similar to those of the previous research.

Lee et al. also compared the Bone Level versus Tissue Level connections within the same implant system when subjected to load in a simulation study. Very significant results were obtained (*p* < 0.001), presenting better connection behavior at the tissue level than the connection at the bone level when subjected to load, with the latter presenting a more significant presence of microgaps [28].

In addition to the type and design of the connection used, there are other parameters related to the connection that can influence the stress experienced by the implant and potentially lead to its fracture. These include the axial axis of the implant under load, unitary situations versus multiple splinted restorations, and the prosthetic design used in the restoration [12,13,24]. In the case of the most vulnerable restorations, which could be said to be single-tooth restorations, Poovarodom P et al., in their study, found that the height of the Ti-Base abutment was a determining factor in the concentration of stresses, indicating that a greater gingival height behaved better biomechanically compared to a shorter one [13]. In the case of single-tooth restorations, Poovarodom P et al. in their study found that the height of the Ti-Base abutment was a determining factor in the concentration of stresses, indicating that a greater gingival height behaved better biomechanically compared to a shorter length.

In short, although it could be said that the study’s hypothesis is confirmed, the conical connection seems to be the most reliable connection from a biomechanical point of view. According to the literature review carried out by the authors of this study, this type of connection allows for a greater distribution of the forces that affect the implant connection and those transmitted to the surrounding bone tissue [15,17,19]. However, it is also true that different designs of this type of connection exist (degree of taper, length of the cone, association with another geometric element, etc.). In addition, it is difficult to find studies comparing implants with the same geometry and materials, where the only differentiating element is the type of connection [21,23,25].

The latter is considered an important source of bias since other elements coexist in the design and manufacture of the implant. The evaluation of the role of the connection design may be altered by other parameters present in the implant or by differences between two implants being compared [15,16,20,29]. These aspects, as well as the heterogeneity in the design of the studies included in the review, can influence the conclusions drawn, as multiple variables can affect the final result.

As a limitation of this study, it should be noted that only studies carried out in vitro have been analyzed. This is because the objective was to reach a purely mechanical conclusion regarding implant fatigue. However, it should be added that after this type of testing, in vivo studies should be conducted, as they influence another series of variables in these circumstances.

For all the above reasons, the authors of the present study suggest that there could be interesting future lines of research in which different types of conical connections (length of the cone, degrees of inclination, etc.) could be compared to determine the ideal design that performs best under load, as long as it is the only differentiating element, establishing a single body of the standard implant. Furthermore, all the connections analyzed should be manufactured from the same material. Additionally, it would be interesting to study the relationship between the implant connection associated with other geometric elements of the implant, as well as different materials (coil design, thickness, manufacturing material, etc.), to determine the most optimal relationship with the load and thereby establish an ideal design from the biomechanical point of view.

Finally, it should be noted that it would be interesting to incorporate studies that analyze the long-term biomechanical resistance associated with different types of implant connections. Although, as mentioned above, another series of parameters would come into play.

## 5. Conclusions

Within the limitations of this study, it can be concluded:

1. The design of the connection seems to play an important role in the fatigue resistance of the implant under load, concentrating stress to a greater or lesser extent at a given point, depending on the design of the connection.
2. The conical connection appears to be the one that performs best in the different biomechanical situations that may arise. However, no specific design appears to be superior to others.
3. In compromised situations, such as short implants, it would be interesting to use a Tissue Level connection design. This type of connection seems to perform better under load.
4. More tests must be carried out to establish a type of conical connection design that is more optimal for the different biomechanical situations. In addition, these studies must be carried out without other geometric or implant elements that could introduce bias.

## Figures and Tables

**Table 1:** The results table shows the articles analyzed in the review, the sample used in each study, and the data of interest related to the present study. Legend: TL: Tissue Level; BL: Bone Level.

Authors	Journal	Type of Study	Type of Connection/s	Diseño estudio/ Conexión Analizada	Noteworthy Fact(s)
Zieliński R et al. [12].	Materials (Basel).	In vitro study	Hexagonal Internal	Biomechanical Analysis of a 3.35 Internal Hexagon Connection Implant (INTEGRA OPTIMA^®^)	The axial axis of the implant is related to the direction of forces, which is a key factor. Internal hexagon angle zones are critical zones. Fatigue resistance was determined at a F = 200–210 N level.
Poovarodom P et al. [13].	J Prosthet Dent.	Simulations Study	Morse Cone	Implant with a Morse taper (Astratech^®^; Dentsply-Sirona^®^), with different gingival heights on the abutment	A TiBase abutment with a higher gingival height reduced stress magnitude.
Alberti A et al. [14].	J Oral Implant.	In vitro study	Conical	Comparative study: Narrow 2.9 mm and 3.3 mm diameter implants with tapered connection (Conical)	The fatigue limit was recorded at 220 (2.9 mm) and 240 N (3.3 mm). No significant difference.
Byun SH et al. [15].	Bioengineering (Basel).	Simulations Study	Conical	Comparative study: AnyOne^®^ and BlueDiamond^®^ Implants (MegaGen^®^) (conical connections)	Connection stability is a critical biomechanical factor.
Giner S et al. [16].	Materials (Basel).	In vitro study	Tube-in-tube Conical	Comparative study: Implant with tube-in-tube connection and conical connection rehabilitated with monolithic crowns	The mechanical performance of the internal conical connection was superior to that of the tube-in-tube connection. (*p* < 0.05)
Lemos CAA et al. [17].	J Oral Implant.	In vitro study	Hexagonal Internal Hexagonal Internal Morse Cone	Comparative study: External hexagonal connection vs. Morse Taper Cemented vs. bolted crowns Metal-ceramic vs. monolithic crowns	The Morse cone exhibited lower microstrain values (range: 1295–1432 με) compared to the external hexagonal connection (range: 1832–2715 µε). The retention system did not affect microstrain in cortical bone tissue under both loads. No differences between metal-ceramic and zirconia monolithic crowns were observed regarding microstrain and stress distribution.
Araki H et al. [18].	Int J Implant Dent.	In vitro study	TL BL	Comparative study: Biomechanical analysis between standard and short implants with TL ^1^ and BL ^2^ connection of pure titanium and titanium-zirconia	When the implant body length must be shorter, TiZr ^3^ and a TL connection design may be a better mechanical choice than pure titanium and a BL ^2^ connection.
Lee H et al. [19].	J Prosthetic Dent.	In vitro study	TL TL Wide BL Internal Bl External	Comparative study: Biomechanical analysis of short implants in 4 connection types (TL ^1^, TL ^1^ Wide, BL ^2^ Internal, BL ^2^ External)	The BL ^2^ internal connection abutment showed higher stresses in the implant components. TL ^1^ had better results.
Prados-Privado M et al. [20].	Med Biol Eng Comput.	In vitro study	External Hexagon	Biomechanical analysis external connection implant 3.5	Adequate biomechanical behavior over the reference values.
Yao KT et al. [21].	J Oral Implant.	Simulations Study	Conical	Biomechanical simulation analysis of conical connection with different grades (Ankylos System)	The optimal design (based on the Ankylos system) was a 10.18° cone.
Bordin D et al. [22].	J Mech Behav. Biomed Mater.	In vitro study	Conical	Comparative study: Biomechanical analysis of 2.9 mm vs. 3.3 mm conical connection implants.	Fatigue tests up to 180 N (both connections). No significant difference. Complications were reduced to abutment fracture.
Wang K et al. [23].	Mater Sci Eng C Mater Biol Appl.	In vitro study	Conical	Comparative study: Biomechanical analysis of conical connection with five types of taper degrees.	Significant differences in the degree of taper and implant strength.
Flanagan D et al. [24].	J Oral Implant.	In vitro study	Conical	Biomechanical strength of the conical connection under load	Axial loading does not prove to be a problem. Oblique loading may compromise the implant’s resistance to fracture.
Balik A et al. [25]	J Oral Implant.	Simulations Study	Internal hexagonal External hexagonal Tube-in-tube Conical Conical with the internal hexagon.	Comparative study: Evaluation of 5 types of connection of different implant brands. Connections: Internal hexagonal, external hexagonal, tube-in-tube connection, conical, and conical are associated with the internal hexagon.	The conical connection associated with an internal hexagon presents the best results.
Liu S et al. [26]	J Prosthetic Dent.	Simulations Study	Internal Conical	Comparative study: Analysis between 2 types of connections. One with a change of platform and one without a change of platform.	The platform change seems to behave better under load than the regular platform.
Freitas-Júnior AC et al. [27]	Dent Mater.	In vitro study	Hexagon Interior External hexagon	Comparative study: Evaluation of internal vs. external hexagonal connection with and without platform change.	Changing the platform in the internal connection seems to improve fatigue resistance, but not in the external connection.
Lee H et al. [28]	Comput. Methods Programs Biomed.	Simulations Study	BL TL	Comparative study: BL vs. TL associated with different variables (load types, implant diameter, etc.)	Better loading behavior of the TL implant compared to BL. BL connection presented a more significant presence of microgaps than TL under load (*p* < 0.001).
De Paula GA et al. [29]	Implant Dent.	Simulations Study	External hexagon Conical	Comparative study: External connection vs. internal connection to the load.	Better performance and lower failure rate of the internal connection compared to the external connection.
Mitra D et al. [30]	J Dent Res Dent Clin Dent Prospects.	Simulations Study	Hexagon Interior Three channels Conical	Comparative study: Load behavior of three types of connections with and without platform change: hexagonal internal, three-channel internal, and conical internal.	Better results in conical connection with platform change.

^1^ TL: Tissue Level. ^2^ BL: Bone Level. ^3^ TiZr: Titanium-Zirconium Alloy.

## Data Availability

No new data were created or analyzed in this study.
